# Impact of Elimination or Reduction of Dietary Animal Proteins on Cancer Progression and Survival: Protocol of an Online Pilot Cohort Study

**DOI:** 10.2196/resprot.5804

**Published:** 2016-07-29

**Authors:** Anna Catany Ritter, Annarita Sabrina Egger, Jennifer Machacek, Rosa Aspalter

**Affiliations:** ^1^ Association for Research and Support of Health Promoting Nutrition during Cancer Disease Vienna Austria

**Keywords:** cancer, neoplasms, diet, vegans, vegetarians, omnivore, remission, disease outcome

## Abstract

**Background:**

Current evidence suggests that the incidence of cancer is low in vegan populations, and experimental studies have revealed a significant role of dietary proteins in cancer development and progression. However, little data currently exists regarding the effect of a plant-based diet on the progression of diagnosed cancer.

**Objective:**

The main objective of this study is to determine if a reduction or total elimination of animal protein from the diet can positively influence the outcome of an existing cancer and, in addition to standard oncological therapies, increase remission rates.

**Methods:**

The primary aim of this online study is to test the effect on remission rates in cancer patients (primary outcome) with distinct self-selected dietary patterns (omnivore, lacto-ovo-vegetarian, vegan), and allow for an estimation of the effect size. Secondary outcomes are tumor behavior, relapse-free interval, therapies, therapy tolerability and side-effects, comorbidities, medication, quality of life, acceptance, and feasibility of the selected diet. Safety concerns exist for vegan diets (especially in cancer patients) and the study will carefully monitor for deterioration of health, tumor progression, or malnutrition. Furthermore, the study will evaluate the online portal as a study platform (technical and safety aspects, and sequence of displayed questionnaires) as well as the validity of self-reported and online-generated data.

**Results:**

The study was performed between April, 2015 and June, 2016, and a preliminary evaluation of safety aspects was undertaken after June, 2016. Primary and secondary outcomes will be evaluated when the final patients complete the study in December, 2016.

**Conclusions:**

This study will reveal information about the effects of dietary patterns on cancer disease and progression. The methodology of the study addresses several aspects and limitations of nutrition studies in cancer patients, such as precision of nutrition data, acceptance criteria, online methodology, and safety aspects.

**ClinicalTrial:**

Clinicaltrials.gov NCT02437474; https://clinicaltrials.gov/ct2/show/NCT02437474 (Archived by WebCite at http://www.webcitation.org/6jL7UUCVq)

## Introduction

Every year, cancer is diagnosed in 14.1 million people worldwide, and 8.2 million people die from this disease. These figures represent 13% of all deaths around the world, and malignant tumors are one of the main causes of death in industrialized countries [1]. The prognosis for the future is even worse, with a 70% increase in cancer incidence expected, meaning 22 million new cases will be diagnosed every year over the next twenty years [1]. The most common types of cancer for men are lung, prostate, and colorectal cancer (CRC), while women mostly suffer from breast, colorectal, and lung cancer [1].

Cancer is known to be a multifactorial disease with many external risk factors that need to be considered, especially lifestyle factors. In recent years, nutrition has been highlighted as a key risk factor, and is now considered to be responsible for 30% of all cancer cases in industrialized countries [2]. Different nutritional aspects have been implicated in the stimulation of a large number of cancers. Alcohol is known for its connection to liver cancer, but high body mass index (BMI) and obesity also play a major role in cancer development [3-5]. While these factors primarily affect industrialized countries, cancer in developing countries is often linked to micronutrient deficiencies [6].

Other cancer risk factors include food that has been prepared at high temperatures, high fat consumption, and the excessive usage of salt. Current World Health Organization (WHO) reports also support the hypothesis that above all, red and processed meats are important carcinogens, partly due to nitrites contained in these foods, along with the smoking process. In contrast to this finding, other nutrition aspects (ie, high intake of fiber, and fruit and vegetables) have a cancer protective effect [7-10].

Studies indicate that cancer is low in vegan populations [11,12], but little data exists regarding the effect of a plant-based diet on the progression of diagnosed cancer. However, evidence suggests that a vegetarian or vegan diet provides many other health benefits, such as lower incidence of high BMIs, obesity, and cardiovascular disease [11,12]. The Second World Cancer Research Fund/American Institute of Cancer recommends a mostly plant-based diet and a reduction of red and processed meat in its Research Export Report of 2008 [13], which is also supported by current reports issued by the WHO in 2015 [14]. However, data referring to the therapeutic setting (ie, patients diagnosed with cancer) are still missing. It is of great scientific interest to test the influence of different dietary patterns on the progression of cancer and cancer survival.

Current treatment options for cancer include surgery, radiotherapy, and chemotherapy [1]. Although international research has made progress in the development of new and more specific therapies, treatment options are still limited [15]. Cancer treatments represent a major financial burden on health care systems, patients themselves, and social systems. The main objective of this study is to determine if a reduction or total elimination of animal protein from the diet can positively influence the outcome of an existing cancer and, in addition to standard oncological therapies, increase remission rates.

Furthermore, we aim to estimate the effect size, and enable sample size calculations for future studies. A small number of studies have concentrated on the effect of specific foods, food groups, or food ingredients, such as the effect of soy product consumption in breast cancer patients [16-19], or the effect of reduced red meat intake in patients with colon cancer [20] and prostate cancer [21]. These results are still inconclusive and often show relatively minor effects, likely due to the fact that these studies investigated isolated foods or food groups and not dietary patterns; this study aims to address this limitation.

Cancer patients receive advice from clinicians and their families and friends with respect to nutrition, but studies indicate that patients do not follow this advice over a significant period of time, and improvements based on dietary advice are limited [22,23]. Therefore, it is necessary to test the acceptance and feasibility of different diets, particularly a plant-based diet, in cancer patients. This study hopes to determine if patients who eat a typical omnivore diet can adjust to an alternative diet, and maintain this lifestyle over a period of 6 months or longer. Concerns exist that suggest a vegan or vegetarian diet could lead to a deterioration of health, to tumor progression, or to malnutrition. To address this issue, this study will carefully monitor any deterioration of the patients’ health status.

Previous nutrition and oncological studies have required many professional staff and enormous financial resources. Such studies are often time-consuming for the patients (therefore limiting compliance), necessitating the development of new tools to simplify the process of data collection. To this end, new technologies have been developed and intensively tested [24]. This study will examine the usage of a new online portal as a study platform, in addition to standard medical practices. If this platform proves to be useful, it could be adapted for future studies in the field of nutritional health care.

The validity and significance of self-reported and online-generated data has been questioned [25,26]. Therefore, it is necessary to test these parameters, along with the usability of online data with regard to future applications of research results.

This study aims to address the following issues: (1) to test the hypothesis that the elimination or reduction of dietary animal protein leads to an improved prognosis in tumor patients, as defined by the remission rates, tumor behavior, and relapse-free interval; (2) to estimate the effect size and enable sample size calculations for future studies; (3) to test the feasibility and tolerance of different diets (particularly a vegan diet) in cancer patients, and to assess the effect of a vegan diet on deterioration of health, tumor progression, and malnutrition; (4) to test the online platform as a study platform; and (5) to test the validity of self-reported and online-generated data.

## Methods

### Study Design

This is a prospective, longitudinal, and observational cohort study of cancer patients with four cohorts: (1) omnivore diet, (2) lacto-ovo-vegetarian diet, (3) vegan diet, and (4) other diets (see Table 1). All four cohorts will complete online questionnaires including a baseline assessment and follow-up assessments at 3 and 6 months (Figure 2). At the 12 and 24 month time points, participants will be contacted via email to complete a truncated study questionnaire.

**Table 1:** Definition of dietary patterns.

Cohort	Meat & fish	Milk & dairy products	Eggs	Honey	Plant products
Omnivore diet	+	+	+	+	+
Lacto-ovo-vegetarian diet	-	+	+	+	+
Vegan diet	-	-	-	-	+
Other diets	Those that do not correspond to any of the above				

**Figure 1:**
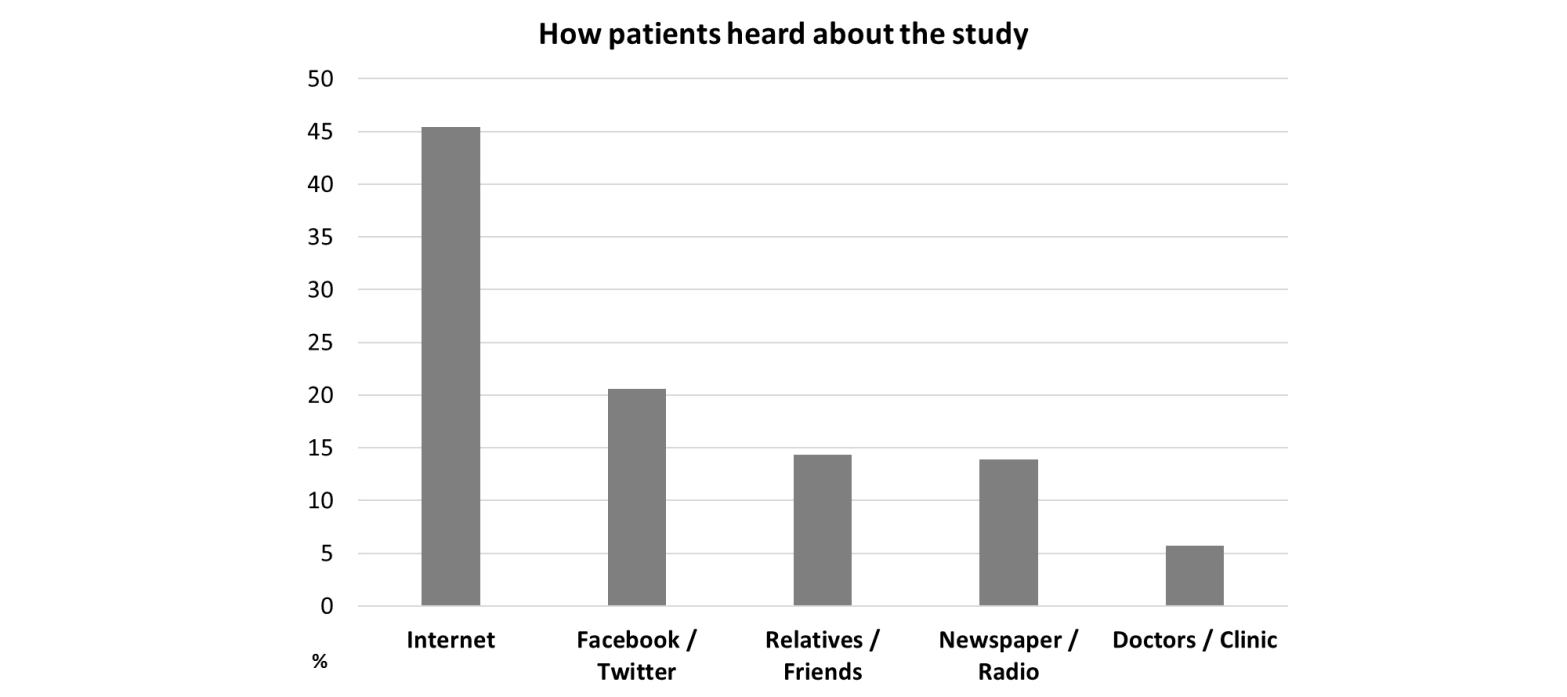
How patients heard about the study (n=209).

### Eligibility and Recruitment

Patients were eligible for this study if they were diagnosed with cancer, >18 years of age, and were included in a tumor treatment regime or a follow-up program. Exclusion criteria included psychiatric treatment during the previous 3 months, pregnancy, breastfeeding, BMI below 18 kg/m^2^, major difficulties with food intake (eg, swallowing, lack of appetite), and participation in other studies requiring the use of a special diet.

Potential participants were recruited online (via the study website, Facebook, online reports, and television and media reports) and offline (via clinical practices, oncological departments, and associations supporting cancer patients). Recruitment took place between April, 2015 and June, 2016.

### Study Population and Setting

Whenever possible, participants selected one of three defined diets (omnivore, lacto-ovo-vegetarian, or vegan) at baseline. If none of these diets was applicable, participants were classified as having *other diets* (Table 1). Participants were instructed to maintain the chosen diet for 6 months. In addition, respondents were encouraged to continue their prescribed cancer treatment during the study period, and to undergo all recommended follow-up investigations.

Calculation of the required sample size for this study is not possible, as the effect size of the dietary patterns is unknown. Therefore, this study is classified as a pilot study.

**Figure 2:**
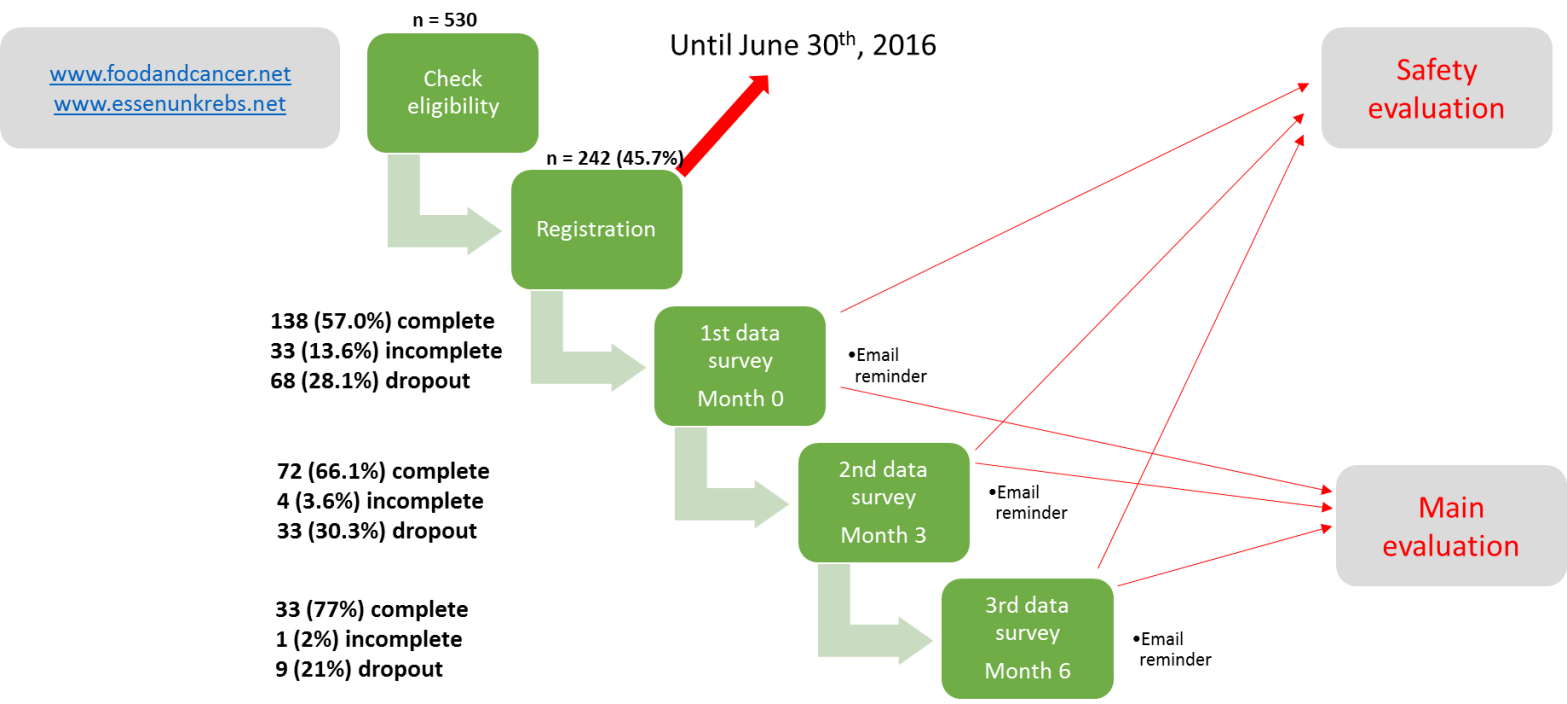
The study protocol showing the 5 steps for each participant, the planned evaluations, and the number of participants and dropouts.

The study is being conducted online via a bilingual study website [27]. Registration processes, eligibility assessments, and questionnaires at baseline and follow-ups are all completed online between April, 2015 and December, 2016. Prior to study participation, potential participants performed an online eligibility check (Multimedia Appendix 1), and were asked to provide contact information and a signed patient information and consent form. After registration, participants received an email including their personal login information for the study website, their study identification number, and a link to their baseline questionnaire. Invitations for assessments at 3, 6, 12, and 24 months are delivered by email (Figure 2). If participants do not fill in their questionnaires at the respected time, they are sent reminder emails. In addition to the three main study questionnaires, study participants are asked to report any severe deterioration, unplanned change of their selected diet, or withdrawal from study participation, via online forms (Multimedia Appendix 2).

Ethical approval was obtained from the Ethics Committee of the City of Vienna on February 2nd, 2015 (EK 15-021-VK_NZ).

### Study Groups

At baseline, participants chose one of three defined diet forms for the study period or *other diet* (Table 1). Definitions and nutritional information regarding the diet forms were compiled by a nutritionist, and are available on the study website. All groups were given clear information regarding the avoidance of nutrient deficiencies, and participants were encouraged to continue their prescribed cancer treatment and follow-up investigations. Participants were also advised to have their serum levels of albumin, iron, vitamin D3, and vitamin B12 checked regularly.

### Measures and Evaluation Procedures

The primary outcome of this study is defined as complete tumor remission, and calculated as the rate of complete remissions at 6 months by intention-to-treat (ITT) analysis.

Secondary outcomes include tumor history, further classifications of tumor behavior (complete remission, partial remission, no change, or progression), relapse-free time (in months), survival rates for end stages, previous and current tumor therapies (including tolerance and side effects), comorbidities (eg, high blood pressure, stroke), as well as medications unrelated to the tumor (including dosage and intake plan). The determination of tumor behavior during the study period will be evaluated by two investigators, each using independent radiological and laboratory reports.

Data regarding previous nutritional history, the chosen diet during study participation, acceptance and feasibility of the chosen diet, frequency of dietary pattern change, extent and duration of nutritional changes, and the type and strength of support for nutritional changes will also be assessed. In addition, during baseline and follow-up surveys, adherence to dietary patterns will be cross-referenced by food frequency questionnaires (FFQs), which were specifically adopted for discrimination of plant- and animal-derived foods. Participants were also asked to self-report any unplanned change in diet during study participation. Nutritional status of participants will be evaluated using body weight (kg), BMI (kg/m^2^), laboratory test results (eg, albumin), and questions referring to eating problems. Results referring to nutrition and dietary patterns will be evaluated in terms of change and duration of change in dietary patterns (in months).

Study questionnaires also contain questions referring to quality of life covering the Karnofsky Index, as well as additional dimensions (ie, psychosocial wellbeing, psychosocial support). Online questionnaires were designed to be self-instructive and self-evident (Multimedia Appendix 3). In order to evaluate these parameters, questions pertaining to the study platform and interaction with the study team were also included in the questionnaires. The list of parameters included in the study questionnaire was designed to examine all study objectives, and is detailed in Multimedia Appendix 4.

### Confounders and Bias

Possible confounders include gender [28] and BMI [29]. Moreover, it is likely that tumor stage and type may influence the results of this study. Therefore, we will examine these factors and adjust our interpretations accordingly. Furthermore, the nature of our website (which highlights the benefits of vegetarian and vegan diets) might attract a population that actively seeks healthy lifestyle habits [27]. Conversely, this group might prefer alternative therapies, and be less compliant to conventional oncological therapies. Therefore, we plan to undertake a subanalysis for treatment groups (oncological treatment followed, oncological treatment rejected, or oncological treatment completed).

### Nutrition Safety

To ensure timely detection of the deterioration of nutritional and/or health status of the patients in this study, an intermediate data evaluation is planned, and will include all participants registered before January, 2016. This evaluation will examine tumor staging and the nutritional status of all participants. If this evaluation demonstrates a significantly increased risk of health deterioration in one diet group compared to the omnivorous group, the study will be stopped preterm.

### Feasibility, Physical Tolerance, and Acceptance of Chosen Diet

Feasibility of the protocol will be examined by (1) assessing compliance with the chosen diets at the 3 and 6-month time points, (2) asking the study participants to report any intermediate changes of the planned diet, and (3) specific questions contained in the questionnaire regarding the ease or difficulties of the diet, and support from family members and medical staff (Multimedia Appendix 4).

Physical tolerance will be examined via questions regarding physical symptoms. Acceptance will be examined via questions regarding ease, difficulties, and willingness to continue their diet until the study is completed and thereafter (Multimedia Appendix 4).

### Online Platform Performance

Prior to the recruitment phase, the study platform (checklist, registration) and surveys were tested by four patients, and adaptations were made according to their feedback. As shown in Multimedia Appendix 4, the performance of the online platforms (webpage platform, survey platform) was assessed via questions regarding users’ experience with the platforms, and with the questionnaires (eg, technical performance, ease of understanding the questionnaires, significance of content). Moreover, it was possible to evaluate time spent filling in the questionnaires, dropout rates between and within the questionnaires (incomplete questionnaires), webpage user statistics, and user characteristics such as time spent reading web content, favorite web content, and user interactions.

### Nutrition Data Validity

As shown in Multimedia Appendix 4, self-selected dietary patterns will be cross-referenced via FFQ. Moreover, laboratory parameters will include albumin, urinary pH, blood urea nitrogen, and hematocrit. It was not possible to control for additional vitamins, such as vitamin C. Questions regarding motivation and intention to choose a certain dietary pattern will serve as data to further support the validity of the trial, or reveal potential inconsistencies.

### Data Safety

Online data safety was a priority in the development of this study, and we strictly separated participants’ personal data (used for communication) from anonymous health/disease related data. To ensure anonymity, each participant received a personal login and study identification number. Administrative (personal) data were collected and stored on servers with enhanced security features. Password-restricted logins and Secure Sockets Layer-encrypted data transmission were used, and host server providers were required to sign a contract according to the German Bundesdatenschutzgesetz [30] to guarantee state-of-the-art handling, storage, and deletion of personal data. Health-related data were collected and hosted at Survey Monkey [31], using anonymous study questionnaires on a server compatible with the Health Insurance Portability and Accountability Act.

### Statistical Analyses

Statistics regarding safety issues will be performed in July, 2016 and a complete evaluation will be performed in January, 2017. Comparison of dietary groups in regard to primary and secondary end points will be performed as ITT. Data will be analyzed and adjusted for possible confounders (eg, gender, age, tumor type, tumor stage, weight status, accompanying treatment) and stratified by sex, age, type of tumor, tumor staging, and oncological therapy. Changes over time (eg, weight changes) will be analyzed by paired sample tests. Unpaired tests will be used when making comparisons between cohorts. Descriptive statistics will be used to summarize parameters such as comorbidities, oncological therapies, and additional therapies. *P*-values of <0.05 will be considered significant.

## Results

Data collection commenced in April, 2015 and recruitment ended in June, 2016. After completion of the study in December, 2016, data will be analyzed and prepared for publication.

### Recruitment and Dropout Rates – Preliminary Experiences

As of May, 2016, 530 participants filled out the checklist and 242 (45.7%) have registered for the study. Most respondents were female (174/242, 71.9%), while 68 were male (28.1%). Mean age was 54.7 years (standard deviation 11.7). Most participants were recruited in German speaking countries (Germany, Austria, and Switzerland). As shown in Figure 1, most participants found the website via the Internet or social media (eg, Facebook and Twitter).

Dropouts occurred at each stage of the study, from the checklist for eligibility to the third data survey, as shown in Figure 2. After filling out the registration forms, 242 participants received the invitation for their first data survey, which was completed by 138 respondents (57.0%). Some respondents (33/242, 13.6%) sent an incomplete survey, and 68 of the original participants (28.1%) did not send a survey. Dropout rates consider participants who withdrew study participation, died, or did not answer after being reminded by email. Four patients have been reported to have died as of May, 2016.

Three months after completing the first survey, participants received an invitation for the second survey (n=109 as of May, 2016), which was completed by 72 respondents (66.1%). Four participants (4/109, 3.6%) sent an incomplete survey and 33 (30.3%) dropped out of the study at this point. The study is still underway, and participants who did not receive the second and/or third surveys have been excluded from these calculations.

The 6-month data survey was sent to 43 participants who completed the first and second surveys. As of May, 2016, this survey was completed by 33 participants (77%); 2% (1/33) sent an incomplete survey, and 21% (9/33) dropped out of the study at this point. Given the number of participants who enrolled in the study at least 6 months before May, 2016 (n=101), this gives a completers rate of 32.7%.

### Preliminary Experiences with Online Questionnaires and Study Platform

**Figure 3:**
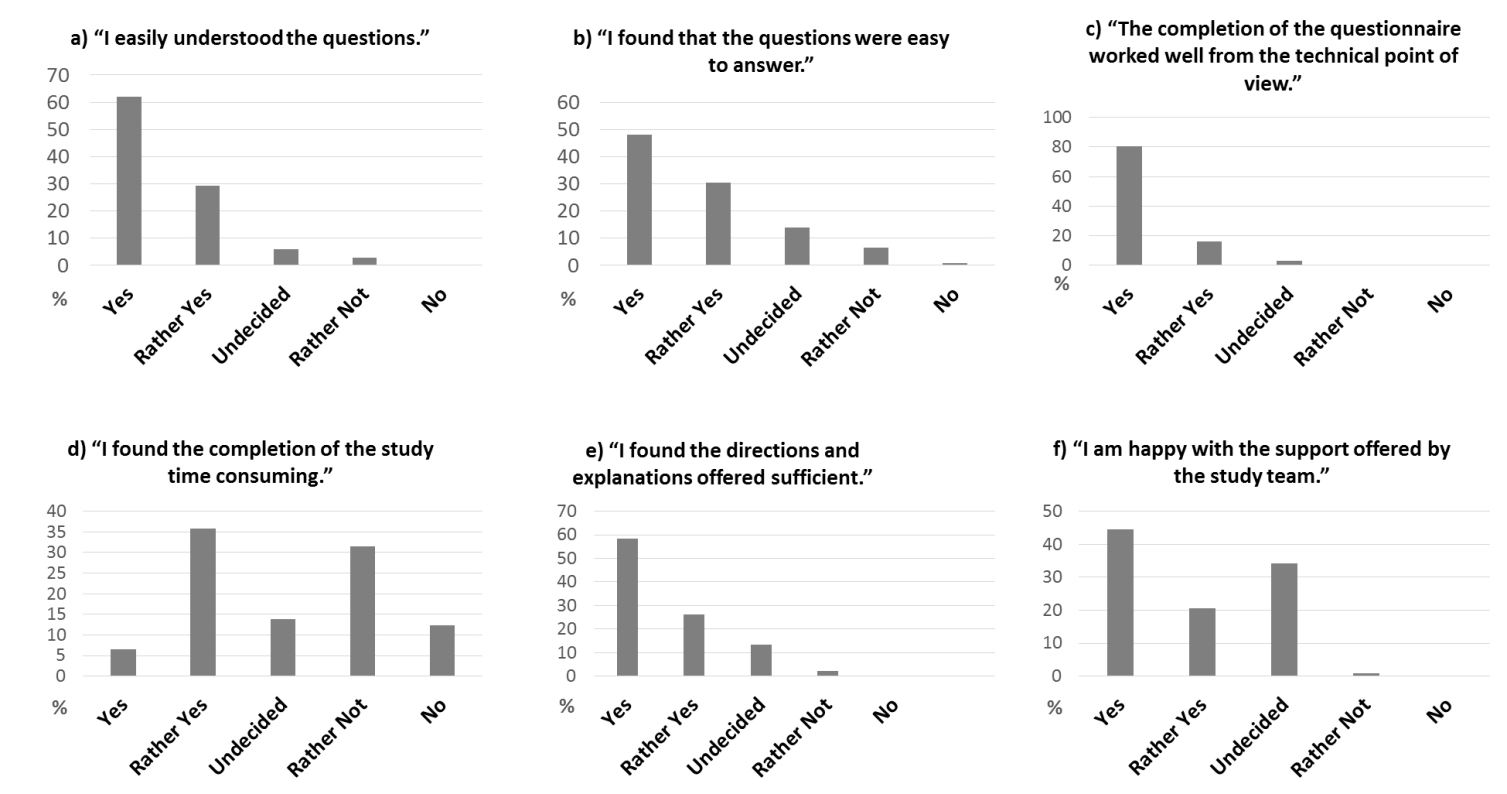
The participants’ opinions about data surveys (n=138).

The questionnaires in this study are very comprehensive (more than 100 items) and contain questions referring to medical data. Participants are also asked about the experience, acceptance, and usability of the study platform. The data collected from the first study survey (n=138) are displayed in Figure 3. The overall acceptance of the questionnaires was good, and most (62% *yes* and 29% *rather yes*) of the participants found the questions easy to understand (Figure 3 a), while 78% (48% *yes* and 30% *rather yes*) found them easy to answer (Figure 3 b). Almost all participants (80% *yes* and 16% *rather yes*) had no problems with the technical aspects of the study (Figure 3 c). When asked if the questionnaire was too time consuming, an equal number of participants (43%) answered with *yes* or *rather yes*, compared to participants who answered with *no* or *rather not* (Figure 3 d). Most (58% *yes* and 26% *rather yes*) of the participants felt that the directions and explanations were satisfactory (Figure 3 e), and the support of the study team was adequate for most (44% *yes* and 21% *rather yes*) of the participants (Figure 3 f). Many participants never contacted the study team (apart from study registration), resulting in 34% of the participants rating this aspect as *undecided*.

## Discussion

The purpose of this study is to assess whether a reduction or elimination of animal proteins from the diet influences the course of existing cancer, and if this tactic can increase remission rates while following standard oncological therapy. Data is being collected using online data surveys.

### Study Aims and Approach

To date, only a small number of studies have investigated the effect of nutrition on established cancer. Among these studies, research on CRC, prostate cancer, and breast cancer have given insights regarding the impact of animal proteins on cancer [16,18,20,21]. McCullough et al found higher CRC-specific mortality in patients with consistently high intake of red and processed meat pre- and post-diagnosis, in comparison to those who did not [20]. Interestingly, the difference was not significant if red and processed meat intake were calculated separately. This result could indicate the significance of the total amount of animal proteins and the importance of defining food patterns. This study did not examine a group without meat consumption, possibly reducing the effect size on CRC-related mortality [20].

Richman et al reported that the post-diagnostic consumption of processed and unprocessed red meat, fish, or skinless poultry was not associated with prostate cancer recurrence, whereas consumption of eggs and poultry with skin may increase the risk of recurrence [21]. The FFQ used in this study included 137 food and beverage items and supplements, from which the research team selected five food groups (processed red meat, unprocessed red meat, fish, poultry, and eggs) but did not include dairy products in their analysis. Current research suggests that the intake of dairy products is linked to prostate cancer [32,33], and this finding may have affected patient cancer recurrence rates in the Richman et al study. In another study, researchers observed that men with a high post-diagnostic cruciferous vegetable intake had a statistically significant (59%) decreased risk of prostate cancer progression compared to men in the lowest quartile [34].

It has been demonstrated that breast cancer patients who consumed high levels of soy products had cancer recurrence rates equal to (and in some cases even less than) patients with low consumption [16,18], but it was not reported whether the patients consumed meat, fish, eggs, or milk, and in what quantities. Similarly, Pierce et al reported that breast cancer patients who continued consuming meat, but increased their intake of fruits and vegetables to five portions per day and physical activity to six times per week, had increased survival rates by as much as four fold, in comparison to the lowest intake and physical activity cohort [35].

Animal proteins have been reported to play a significant role in certain cancer types, such as hepatocellular carcinoma of hepatitis-induced carcinoma [36], and may also be a factor in many other cancer types [37-40]. This study aims to approach all cancer types in the pilot study. In addition, we will not examine single foods or food groups, but instead investigate whole dietary patterns. As we divide our study collective into four cohorts (omnivore, lacto-ovo-vegetarian, vegan, or other), we hope to draw correlations between the amount of consumed animal proteins and cancer progression and survival.

This study will make it possible, for the first time, to estimate the effect size of animal protein consumption in cancer patients, and to stratify for age, body weight, tumor stage, and tumor type. These findings may be of high importance for the selection of certain patient groups for future studies with more detailed questioning.

It is known that dietary advice is difficult to follow for many patients, making it challenging to achieve improved prognoses by dietary advice alone [22,23]. Therefore, it is crucial to examine the acceptance and feasibility of the recommended diet. To this end, tools are needed that can increase motivation and compliance, and reduce attrition rates. Online tools like ours appear to be particularly suitable for this issue, based on a study that used a similar procedure [24].

Changing one’s diet to a more plant-based diet has not only been shown to decrease the negative side effects of chemotherapy in cancer patients [6] (such as the severity of vasomotor symptoms [41] and events of hot flush (HF) in HF-positive women recently treated for breast cancer [42]) but also to regulate weight [12], which plays an important role in the outcome of cancer treatments [43,44]. Suggestions that a vegan diet might lead to deterioration have not be empirically proven [45], and intermediate fasting did not have detrimental effects on patients receiving chemotherapy for hematological diseases [46,47].

Self-reported measures have been frequently used and analyzed in psychological studies in recent years [48], but not as extensively in clinical studies. In the context of this study, we developed and tested an Internet study platform, which enabled us to reach a wider population by decreasing the threshold of participation for patients. In addition, the expenditures for both the study team and the patients are clearly decreased. We hope to use the current study to adapt and optimize the portal for future investigations, including study questionnaires and data-collecting processes. The current study platform may allow for more economical and more precise studies regarding the effects of nutrition on existing cancer.

In contrast to statements that self-reported and online-collected data show less validity, publications exist that demonstrate the opposite [25,49,50]. These new technologies have the potential to increase data precision, quality, and quantity [25,49,50], particularly if the technology of this study platform is integrated with tools for precise dietary monitoring in the future.

### Preliminary Experiences

The preliminary dropout rate of 67.3% (67/101) appears to be quite high. However, this rate is comparable with other studies performed online, in which dropout rates range from 33% [49] and 34% [51], up to 82% [52] and 88% [53], depending largely on the type of study and if participation was associated with clinical contact and intervention. Dropout rates are also influenced by the disease and stage being investigated.

The questionnaires in this study are very comprehensive (more than 100 items) and contain not only personal questions about symptoms and habits, but also questions referring to medical data. It was important to evaluate whether or not participants were able to complete the surveys. The answers regarding experience and usability of the study platform and questionnaires were encouraging, and the majority of participants did not experience difficulties. This notion is supported by the fact that many more registered participants dropped out between the three study surveys than those who left an incomplete questionnaire. Dropout rate was highest after the first data survey and decreased significantly with each subsequent survey. We therefore conclude that anonymity and lack of contact with the study team caused the attrition rates.

### Bias

This study was performed under free-living conditions, and it was not blinded (open-label design). Study participants chose their dietary pattern by themselves, resulting in participants and study team members being aware of the participants’ dietary pattern. This factor could introduce performance bias (systematic differences between groups in the care that is provided, or due to exposure to factors other than the factor of interest). When choosing their dietary pattern, participants were not manipulated in any way to choose a certain group, and we did not declare the vegan diet as a sufficient treatment for cancer, which might have led to exaggerated expectations.

As of July, 2016, 10 participants with cancer diagnosis and treatment were recruited via a clinical practice, and agreed to participate in the control group (unchanged omnivore diet). These participants have personal contact with study team members, unlike the rest of the included participants. However, all four groups (omnivore, lacto-ovo-vegetarian, vegan, or other) received the same neutral recommendations for avoiding malnutrition.

We did not exclude certain cancer types or stages, and cannot exclude a bias towards certain cancer types prior to the onset of the study. The distribution of these factors (frequency of certain types and stages of cancer) will be analyzed in the complete study evaluation.

Participants were recruited from various countries, and we considered socioeconomic and gender aspects in our study websites, newsletters, questionnaires, and dietary recommendations. Finally, a possible limitation of a web-based study is the attrition phenomenon, which leads to attrition bias. We aimed to minimize this effect using email reminders, and by offering frequent newsletters, blogposts, articles, and a Facebook site for the study.

### Conclusions

To date, most studies addressing the topic of nutrition and cancer have only considered the preventive aspect, while our study focusses on the influence of a plant-based diet on an existing cancer. The results of our study will add useful information to currently existing experimental data, and will indicate whether it is possible to influence the course of an existing cancer by changing dietary patterns, such as animal protein consumption.

With our innovative study design that includes online questionnaires, an Internet platform, dietary recommendations and motivating newsletters, we aim to demonstrate a cost-effective technique that can be used to carryout future studies on this topic.

## Appendix

Multimedia Appendix 1 The checklist, which serves for potential participants to check their eligibility.

Multimedia Appendix 2 The login area, which can be accessed by the participant via a username and password. Participants can check their study status, fill out forms for unplanned changes in diet or health status, withdraw from the study, or upload new medical reports.

Multimedia Appendix 3 Example page from study questionnaire one demonstrating guidance of users with predefined answer options.

Multimedia Appendix 4 Data obtained at baseline and follow-ups (months 3 and 6).

## Abbreviations

BMI: body mass index
CRC: colorectal cancer
FFQ: food frequency questionnaire
HF: hot flush
ITT: intention-to-treat
WHO: World Health Organization
