# Supplementary material for: Impact of Elimination or Reduction of Dietary Animal Proteins on Cancer Progression and Survival: Protocol of an Online Pilot Cohort Study
Source: JMIR Res Protoc. 2016 Jul 29;5(3):e157. doi: 10.2196/resprot.5804 (PMC4982911; doi:10.2196/resprot.5804)
Supplement: Multimedia Appendix 1 [file resprot_v5i3e157_app1.pdf]

Checklist for Participation in the Study

Check out if you or your relatives meet the requirements for participation in the Food-and-Cancer-Study!

(All answers have to be made referring to the patient and not referring to the person who collects data.)

Inclusion Criteria

1. There is a history of cancer disease.	yes ☑	no ☐
2. Therapies and / or follow-up examinations are done regularly.	yes ☑	no ☐
3. Age is 18 years or more.	yes ☑	no ☐
4. There is willingness to submit 3 extensive data surveys during the study.	yes ☑	no ☐
5. There is willingness to report changes (e.g. in nutrition habits) during the study (also online).	yes ☑	no ☐

Exclusion Criteria

1. The patient is currently pregnant.	yes ☐	no ☑
2. The patient is breast feeding at the moment.	yes ☐	no ☑
3. Psychiatric treatment was required during the last three months.*	yes ☐	no ☑
4. My Body Mass Index (BMI) is below 18,5 kg/m2 (see below).	yes ☐	no ☑
5. There are great difficulties with eating (diffculties swallowing, loss of appetit,...) leading to reduced food intake.	yes ☐	no ☑
6. Participation in another study which requires adherence to certain nutrition rules.	yes ☐	no ☑

*) A "psychiatric" treatment is not a "psychologicical" or "psychotherapeutic" intervention. An example of a psychiatric disorder is e.g. schizophrenia, expamples of psychological conditions ar burn out or depressive mood. These later do not exclude you from participation.

You fullfill all requirements to participate in the study! Want to register for the study?

SUBMIT AND GO TO STUDY REGISTRATION! RESET

BMI-Calculator

Body size in cm

Body weight in kg

compute BMI

Your Body-Mass-Index

Languages

English
Deutsch

Support and donate!

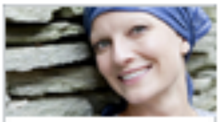

Food and Cancer

Donations: 44

9%

Donate now

betterplace.org

Login for Study Participants

Username

Password

Log In Remember Me

Lost your password?

Information:

Frequently Asked Questions (8)

Nutritional Recommendations for Participants (7)

Scientific Background (2)

Tipps for Relatives and Friends (1)

What study participants say (8)

Blogroll

If you would like to share your experience with cancer and especially with a switch towards
