# Supplementary material for: Impact of Elimination or Reduction of Dietary Animal Proteins on Cancer Progression and Survival: Protocol of an Online Pilot Cohort Study
Source: JMIR Res Protoc. 2016 Jul 29;5(3):e157. doi: 10.2196/resprot.5804 (PMC4982911; doi:10.2196/resprot.5804)
Supplement: Multimedia Appendix 2 [file resprot_v5i3e157_app2.pdf]

My Status

My Study-ID:

✓ Study registration successful on 18-12-2015

✓ Patient information read and accepted on 18-12-2015

1. Data survey due on: 25-12-2015
1. Data survey completed on: 19-12-2015

2. Data survey due on: 18-03-2016
2. Data survey completed on: 08-04-2016

3. Data survey due on: 18-06-2016
3. Data survey completed on:

Sent Reports:

Following reports have been sent to the "Food and Cancer"-study team:

Änderung der Ernährungsweise gemeldt am: 14-03-2016
Übermittlung von Befunden am: 14-03-2016
Übermittlung von Befunden am: 31-03-2016
Änderung der Ernährungsweise gemeldt am: 31-03-2016
Änderung der Ernährungsweise gemeldt am: 05-04-2016

Forms and Important Documents:

Patient information

Form to submit reports (as add on to the main study questionnaires or in between)

Form to report a change in nutrition habits

Form to report a severe change in health state

Form to report study termination

Contact form

Languages

English
Deutsch

Support and donate!

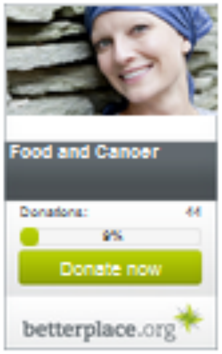

Hello

log out

Information:

Frequently Asked Questions (8)

Nutritional Recommendations for Participants (7)

Scientific Background (2)

Tipps for Relatives and Friends (1)

What study participants say (8)
