# Supplementary material for: Impact of Elimination or Reduction of Dietary Animal Proteins on Cancer Progression and Survival: Protocol of an Online Pilot Cohort Study
Source: JMIR Res Protoc. 2016 Jul 29;5(3):e157. doi: 10.2196/resprot.5804 (PMC4982911; doi:10.2196/resprot.5804)
Supplement: Multimedia Appendix 3 [file resprot_v5i3e157_app3.pdf]

foodandcancer.net

42%

9. Please specify the cell type (histology) of the tumor:

Adenocarcinoma

10. Please specify the "grading" of the tumor:

G2

11. Please specify the "spreading" of the tumor:

T2

12. Please specify the spreading to the lymph nodes:

N0
N1
N2
N3
NX

[illegible]ase specify the stage of Metastasis:

14. Please specify other possible cancer classifications (eg. Dukes 1, FIGO,...):

Prev Next
