# Supplementary material for: Impact of Elimination or Reduction of Dietary Animal Proteins on Cancer Progression and Survival: Protocol of an Online Pilot Cohort Study
Source: JMIR Res Protoc. 2016 Jul 29;5(3):e157. doi: 10.2196/resprot.5804 (PMC4982911; doi:10.2196/resprot.5804)
Supplement: Multimedia Appendix 4 [file resprot_v5i3e157_app4.pdf]

Multimedia Appendix 4. Data obtained at baseline and follow-ups (months 3 and 6).

	**Baseline (month 0)**	**Follow-ups (month 3/6)**
**Regarding inclusion and exclusion criteria**		
Cancer location, tumor type, staging	X	
Time of diagnosis and institution providing the diagnosis	X	
Weight, prev. weight loss, height, BMI, age, sex	X	X
Psychiatric treatment during the last 3 months	X	X
Pregnancy, breast feeding	X	X
**Primary outcome**		
Tumor (re)staging	X	X
Chosen diet during the study (categorical)	X	X
**Secondary outcomes**		
Treatment history	X	X
Current oncological or surgical treatment	X	X
Laboratory testing (especially tumor marker)	X	X
Radiological reports, Computed Tomography Scans	X	X
Therapy tolerability	X	X
Previous diet (categorical)	X	
Previous diet (Food Frequency Questionnaire [FFQ])	X	
Questionnaires regarding quality of life	X	X
Comorbidities	X	X
Current additional therapies	X	X
Lifestyle-factors (Smoking, physical activity)	X	X
Socioeconomic status	X	X
**Questions referring to acceptance and feasibility of the diet**		
Intention for participation	X	
Reason for choosing the respected diet	X	

Questions regarding ease and feasibility of dietary changes		X
Questions regarding support to maintain dietary changes		X
**Questions referring to acceptance and usability of study platform / questionnaires**		
Questions regarding the study questionnaires	X	X
Questions regarding technical performance of study platform	X	X
**Questions to evaluate data validity**		
FFQ	X	X
Control questions in the study questionnaires	X	X
Attending physician or center	X	
Laboratory test to evaluate adherence to diet	X	X
Upload of medical records enabled	X	X
**Administrative data**		
Name	X	
Date of birth	X	
Address, Phone number, email	X	
Contact data of a relative	X	
